# Cutaneous Syncytial Myoepithelioma: A Unique Variant Worth Recognizing

**DOI:** 10.3390/dermatopathology10030030

**Published:** 2023-07-20

**Authors:** Muhammad N. Mahmood

**Affiliations:** Department of Laboratory Medicine and Pathology, University of Alberta Hospital, Edmonton, AB T6G 2B7, Canada; mmahmood@ualberta.ca; Tel.: +1-780-407-2145

**Keywords:** myoepithelial, myoepithelioma, cutaneous syncytial myoepithelioma, cutaneous appendageal tumor, EWSR1

## Abstract

Cutaneous syncytial myoepithelioma is a recently characterized variant of cutaneous myoepithelioma with a distinct histopathological and immunohistochemical profile. It is more common in men and predominately involves upper and lower extremities. Microscopically, it is a dermal tumor with a characteristic solid syncytial growth pattern displaying positivity with EMA and S100 immunohistochemical stains. Lately, EWSR1-PBX3 fusion has been documented in a vast majority. Although it follows a benign clinical course, its histopathological differential diagnosis includes clinically aggressive neoplasia. This contribution summarizes the derivation, clinical presentation, histopathological and immunohistochemical features, molecular genetics, pertinent differential diagnosis, and behavior of this unique cutaneous appendageal tumor.

## 1. Introduction

Benign and malignant myoepithelial neoplasms are well documented in several locations, including salivary glands, breast, and lung. In recent decades, benign myoepithelial proliferations of skin have been increasingly identified and categorized as cutaneous myoepithelioma (CM) [1,2,3]. CM, a dermal tumor formed by proliferating myoepithelial cells, demonstrates morphological diversity and is considered to form a continuous spectrum shared with benign cutaneous mixed tumor (chondroid syringoma) and myoepithelial carcinoma [2].

Within CM, a subset of cases representing a discrete variant designated as cutaneous syncytial myoepithelioma (CSM) has been lately described [3,4,5,6]. While uncommon and recently delineated, this subgroup has a distinctive histomorphological and immunohistochemical profile. In order to achieve precise categorization and avoid mislabeling this lesion as a sinister cutaneous neoplasm, familiarity and knowledge of this variant are essential for residents, dermatologists, and pathologists.

## 2. Origin and Derivation

Myoepithelium or myoepithelial cells are ectodermal-derived modified epithelial cells positioned amid the glandular luminal epithelial cells and the basement membrane. They serve several functions, including contraction in response to cholinergic stimuli to aid the extrusion of glandular secretions, support of secretory cells, and production of basement membrane material. Myoepithelial cells are typically slender and spindle-shaped, possessing cytoplasmic processes which engulf the neighboring epithelial cells. As the name implies (i.e., myoepithelial), they can express attributes of both epithelial and smooth muscle cells, proliferating and differentiating to shape varied morphologies, including spindled, epithelioid, basaloid, plasmacytoid, histiocytoid, and clear cell types [7,8]. Variations such as squamous or chondroid metaplasia can also develop. This diversity in morphology reflects in the heterogeneous microscopic traits of myoepithelial proliferations. They are situated in different body sites, including salivary glands, breast, lung, prostate, skin, and lacrimal glands.

In the skin, they are normally present as an outer discontinuous layer in secretory units of eccrine and apocrine sweat glands, and their existence has been noted in a variety of adnexal tumors [9]. The proliferation of myoepithelial cells is frequently seen in commonly encountered cutaneous mixed tumors [10]. Sharing the same spectrum, CM is formed purely by myoepithelial cells and lacks any tubuloductal elements regularly observed in the cutaneous mixed tumor. Myoepitheliomas are usually considered soft tissue neoplasms; however, in the skin, they are grouped with appendageal tumors. The current World Health Organization classification of skin tumors categorizes them as benign tumors with apocrine and eccrine differentiation subcategories [11]. CM is further categorized into a classical variant and a recently described solid syncytial variant named CSM. The characteristics ascribed to CSM were initially presented by Hornick and Fletcher [3]. Following its initial description, CSM was further characterized in a larger study of 38 cases in 2013 [4].

## 3. Clinical Presentation

CSM is recently recognized, and the actual incidence is not precisely known as only a limited number of cases are reported. It is more prevalent in men (2.5:1, male-to-female ratio), and though cases are seen over a wide age range, they are generally observed in the third to fifth decades of life (median: 39 years) [4]. It occurs predominantly on the upper and lower extremities (74%), including hands and feet; however, cases on trunk and head–neck areas have also been reported. The clinical presentation of CSM is similar to classical CM, yet it is different from a cutaneous mixed tumor which is more frequent in the head–neck area. CSM typically presents as a solitary, painless, dermal, papular/nodular, or polypoid gradually growing lesion with a size range of 0.3 to 2.7 cm (median: 0.8 cm) [4]. Considering the vast array of clinical differential diagnoses offered in reported cases of CSM, the clinical appearance is non-specific, and the diagnosis is almost exclusively reliant on pathological assessment.

## 4. Microscopic Features

**Figure 1:**
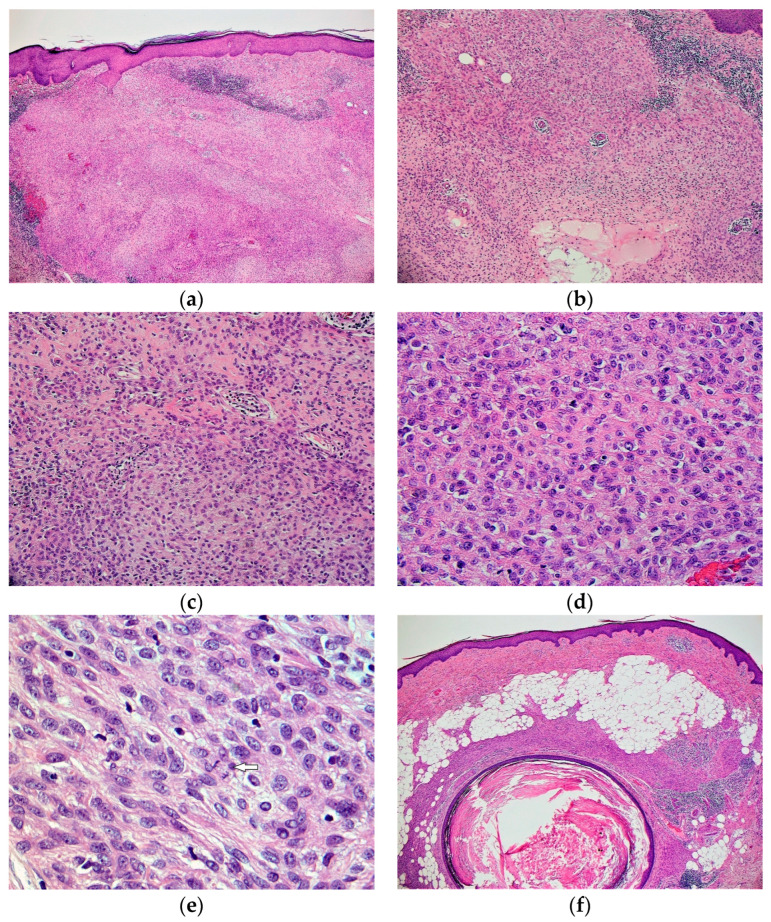
Microscopic features. (**a**) Dermal epicenter of growth which abuts the overlying epidermis. Mild peritumoral lymphocytic infiltrate is also seen (H&E, ×40); (**b**) sheath-like pattern of dermal growth formed by uniform appearing ovoid and histiocytoid cells (H&E, ×100); (**c**) closer view of the solid syncytial pattern formed by uniform monomorphic cells (H&E, ×200); (**d**) vesicular nuclei displaying fine chromatin and nucleoli, surrounded by eosinophilic syncytial cytoplasm (H&E, ×400); (**e**) cellular area shows mitotic activity (arrow) (H&E, ×500); (**f**) adipocytic metaplasia and follicular cyst formation are present (H&E, ×40).

On light microscopic examination, CSM displays a non-encapsulated and non-infiltrative delineated outline of growth. It shows a dome-shaped silhouette with a primarily dermal epicenter of growth. Subcutis, which may be superficially encroached, is not characteristically permeated [3,4,6]. The tumor may abut the overlying epidermis inducing mild secondary hyperplasia; however, no ulceration is recorded (shown in Figure 1a).

The proliferation exhibits a solid sheath-like pattern formed by monomorphic appearing ovoid, histiocytoid, or spindled cells (shown in Figure 1b,c). Short fascicle formation may be present, and multinucleation is generally not seen. The vesicular nuclei demonstrate fine chromatin and distinct small nucleoli, surrounded by eosinophilic syncytial cytoplasm (shown in Figure 1d). They are cytologically bland with no significant hyperchromasia, pleomorphism, apoptosis, or necrosis. The majority of CSM does not display mitotic activity; however, when present, they tend to be sparse ranging from 0 to 4 per 10 HPF (shown in Figure 1e). CSM lacks significant other stromal elements, though fat appearing entrapped in tumors representing adipocytic metaplasia has been repeatedly described (shown in Figure 1f). No ductal differentiation is noted; however, entrapped native skin adnexal elements and occasional small follicular cyst formation are sometimes noticed (shown in Figure 1f). Only rarely, chondroid–osseous differentiation has been focally observed. Associated mild intra-tumoral or peri-tumoral, perivascular infiltrate comprised of lymphocytes, plasma cells, or some histiocytes have been described.

The histopathology of CSM is distinctive and reproducible between tumors. In comparison, analogous to their counterparts in the salivary glands and soft tissue, the classical variant of CM can reveal a range of variable morphologies [5,12,13]. The classical variant is lobulated with reticular, trabecular, solid, or plexiform growth patterns, showing plump epithelioid, plasmacytoid (hyaline cells), spindled or clear cells arranged in cords or nests, and producing/embedded in chondromyxoid, myxoid or hyaline stroma.

## 5. Immunohistochemical Profile and Genetics

Confirmatory immunohistochemical studies are generally required for the diagnosis of myoepithelioma. As myoepithelium can exhibit both epithelial and myoid differentiation, myoepithelioma can show a heterogeneous immunophenotype with a variable combination of keratins, epithelial membrane antigen (EMA), S100, and myogenic markers like smooth muscle actin (SMA) [2,3]. The usual immunolabeling pattern for myoepithelioma is S100, along with at least one epithelial marker. Unlike normal myoepithelium, the proliferating myoepithelial cells in tumors may sometimes lose immunolabeling by myoid stains, causing the absence of SMA expression in a subset of these tumors.

**Figure 2:**
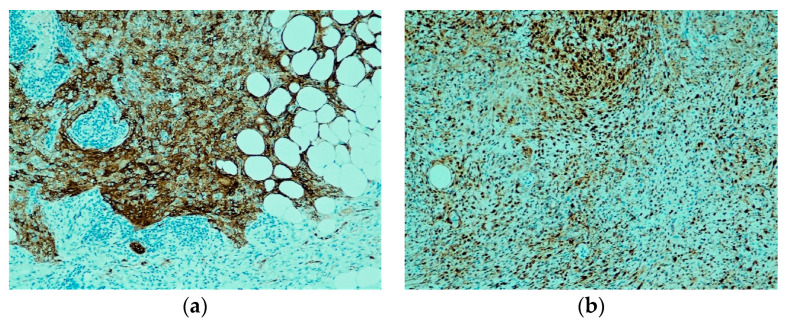
Immunohistochemical stains. (**a**) Diffuse EMA immunohistochemical stain positivity is present (EMA, ×100); (**b**) S100 immunohistochemical stain positivity is present (S100, ×100).

CSM shows a reproducible immunohistochemical profile of EMA and S100 positivity (shown in Figure 2a,b); however, in contrast to myoepithelioma in other sites, most lack keratin expression. In the largest study thus far, diffuse EMA positivity was registered in all 38 of 38 cases (100%) and diffuse S100 positivity in 33 of 38 cases (87%) of CSM [4]. In five cases that were negative for diffuse S100 expression, focal staining was still observed. In a more recent investigation, out of 23 cutaneous myoepitheliomas with syncytial growth, 66% were EMA positive, and 87% were S100 positive [6]. Keratins (including pan-keratin, AE1/AE3, and CAM 5.2) were much less frequent, with negative results in 31 of 36 cases (86%). Glial fibrillary acidic protein (GFAP) stain was variable, exhibiting weak multifocal positivity in 14 of 33 cases (about 42%), SMA was positive in 9 of 13 cases (about 69%), and p63 was expressed in 6 of 11 cases (55%) [4]. Desmin was negative in the majority of cases. SOX-10 expression was shown in 3 of 5 cases (60%) of CM, though these cases were classical rather than syncytial subtypes [14]. Some individually reported cases of CSM do state SOX-10 negativity [15]. HMB-45, Melan-A, and microphthalmia-associated transcription factor (MiTF) were negative [15]. ALK was also negative in these tumors [16]. The classical variant of CM and CSM share the consistent S100 labeling; however, the classical variety showed a lower percentage of EMA positivity (42%) and a higher rate of cases expressing keratins (about 90%) [13].

Ewing sarcoma RNA-binding protein 1 (EWSR1) occurs in up to 45% of skin and soft tissue myoepithelial tumors [5]. Fluorescence in situ hybridization for EWSR1 gene rearrangement has confirmed a very high rate of positivity in CSM (14 of 17 cases, 9 of 9 cases) [4,17]. A novel fusion gene partner has been documented as recent reports disclose EWSR1-PBX3 fusion in a vast majority of CSM [17,18]. Considering the relatively invariable histopathological and immunohistochemical profile of the syncytial variant, the genetic consistency is not unexpected. The importance of gene fusion products is not yet evident in this setting, and they may play a role in the histomorphological appearance of CSM. In comparison, the classical variant of CM has demonstrated several fusion gene partners, and the cutaneous mixed tumor is linked with pleomorphic adenoma gene 1 (PLAG1) aberrations [19].

## 6. Pathological Differential Diagnosis

The histopathological and immunohistochemical profile of CSM generates a broad microscopic differential diagnosis. Due to the syncytial growth pattern, the differential is different from what is usually fashioned for the classical variant of CM. The general category is a superficial dermal tumor and includes both benign and malignant neoplasms [20]. The list of mimickers encompasses lesions belonging to diverse histogenetic groups, including fibrohistiocytic (epithelioid fibrous histiocytoma, juvenile xanthogranuloma, and epithelioid sarcoma), melanocytic (intradermal Spitz nevus, nevoid melanoma, and primary dermal melanoma), peripheral nerve sheath tumors (epithelioid schwannoma, epithelioid perineurioma) and uncertain histogenesis (cellular neurothekeoma).

If a pathologist is not familiar with the CSM morphology, it can pose a potential diagnostic pitfall [21]. Careful inspection of microscopic features and a panel of immunohistochemical stains can aid in establishing the correct diagnosis and rule out clinically significant entities in the differential. Epithelioid fibrous histiocytoma can display the growth of epithelioid cells with eosinophilic cytoplasm in a sheet-like pattern; however, it has collagenous vascularized stroma, shows binucleation, and in general, lacks a spindle-cell component and authentic syncytial architecture. They are also EMA-positive and negative for keratins; however, in contrast to CSM, they are ALK-positive and negative for S100, p63, and GFAP stains [22]. Juvenile xanthogranuloma can mimic CSM, especially if it lacks Touton-type giant cells and lipidization. They are positive for histiocytic markers (e.g., CD68K) and are negative for myoepithelial stains. Epithelioid sarcoma displays epithelioid and spindled cytologically atypical cells in a larger nodular growth [15]. They show deep infiltrative growth, increased mitoses, and central necrosis, sometimes generating a low-power impression of a necrobiotic granuloma. They are also EMA positive; however, compared to CSMs, they express positivity for keratins and CD34 and are negative for S100 and GFAP, and show loss of SMARCB1 (INI1). Cellular neurothekeoma, an uncommon tumor of uncertain histogenesis with suggested fibrohistiocytic or myofibroblastic origin, is formed by epithelioid to spindled cells arranged in a nested or concentric pattern, lacking the syncytial pattern. They are negative for S100 and EMA stains, though they show positivity for S100A6 and CD63 (NKI-C3) markers [23].

S100 positivity in CSM also brings in amelanotic intradermal melanocytic proliferations in the microscopic differential. In particular, a subset of intradermal Spitz nevus can display certain morphological similarities; however, these lesions tend to show nested growth patterns in areas, exhibit maturation upon descent, and may have at least a focal junctional component in the epidermis. Primary dermal melanoma and nevoid melanoma may also enter the differential; however, along with several other differentiating traits, melanoma generally demonstrates a greater degree of cytological atypia and mitotic activity [15]. Although S100 positivity is shared, CSM is negative for other conventional melanocytic markers (i.e., HMB45, Melan-A, and MiTF).

Specific criteria for predicting aggressive or malignant behavior have not been precisely established for cutaneous myoepithelial neoplasms [3]. If criteria validated for myoepithelial neoplasms of soft tissue are employed, the chief criterion for malignancy is cytological atypia, with neoplastic cells exhibiting enlarged nuclei, prominent nucleoli, and coarse chromatin [5]. Cases classified as cutaneous myoepithelial carcinoma or cutaneous malignant myoepithelioma are exceptionally rare and tend to display moderate to severe cytological atypia, high mitotic rate, and necrosis, although cases with a lesser degree of cytological atypia have also been described [24,25]. In comparison, CSM lacks overt cytological atypia, high mitotic count, or necrosis.

## 7. Clinical Behavior

CSM is a benign tumor that rarely exhibits local recurrence. In 21 patients with available follow-up information, only one patient demonstrated local recurrence after about four years [4]. In this patient, the initial procedure was a shave biopsy, and both primary and recurrent tumors did not disclose any cytological atypia, increased mitosis, or additional microscopic traits of malignancy. Six patients with positive biopsy margins displayed no evidence of clinical recurrence, and aggressive clinical behavior or distant metastasis was not seen. Complete conservative excision with negative margins is usually recommended for CSM.

## 8. Conclusions

In sum, CSM is a distinct, recently characterized variant of cutaneous myoepithelioma (Table 1). It is more common in men and frequently occurs on extremities. It displays a syncytial growth pattern, EMA, and S100 positivity, and has shown EWSR1-PBX3 fusion. Due to the relatively recent induction of this appendageal tumor in the dermatological lexicon, dermatologists, pathologists, and residents in training may be unfamiliar with its morphological appearance and immunohistochemical profile. This lack of awareness can lead to the under-recognition of CSM and misdiagnosis as one of its morphological mimics.

## Figures and Tables

**Table 1:** Summary of cutaneous syncytial myoepithelioma.

**Clinical presentation** - Sporadic, solitary, painless, slow-growing, papule or nodule - Men > women, extremities, 3rd to 5th decade, size: 0.8 cm (median)
**Microscopic features** - Dermal tumor with a solid syncytial growth pattern - Uniform, monomorphic, ovoid, histiocytoid, or spindled cells - Vesicular nuclei, small nucleoli, pale eosinophilic cytoplasm - Adipocytic metaplasia, occasional mitosis
**Immunohistochemical profile** - EMA+, S100+, SMA+ - Keratins-, Desmin-, ALK-, HMB45-, Melan-A-
**Genetic profile** - EWSR1-PBX3 fusion
**Pathological differential diagnosis** - Epithelioid fibrous histiocytoma, juvenile xanthogranuloma, epithelioid sarcoma - Intradermal Spitz nevus, nevoid melanoma - Epithelioid schwannoma, epithelioid perineurioma, cellular neurothekeoma
**Prognosis** - Benign, rare local recurrence

## Data Availability

Not applicable.
